# TinyONet: A Cache-Based Sensor Network Bridge Enabling Sensing Data Reusability and Customized Wireless Sensor Network Services

**DOI:** 10.3390/s8127930

**Published:** 2008-12-05

**Authors:** Eui-Hyun Jung, Yong-Jin Park

**Affiliations:** 1 Department of Computer Science, Anyang University / Samseong-ri, Buleun-myeon, Ganghwa-gun, Inchon, 417-833, Korea; E-Mail: jung@anyang.ac.kr; 2 Department of Electronics and Computer Engineering, Hanyang University / Haengdang-dong, Sungdong-gu, Seoul, 133-791, Korea

**Keywords:** Wireless Sensor Networks, Protocol Bridge, Data-centric communication, Virtual counterpart

## Abstract

In recent years, a few protocol bridge research projects have been announced to enable a seamless integration of Wireless Sensor Networks (WSNs) with the TCP/IP network. These studies have ensured the transparent end-to-end communication between two network sides in the node-centric manner. Researchers expect this integration will trigger the development of various application domains. However, prior research projects have not fully explored some essential features for WSNs, especially the reusability of sensing data and the data-centric communication. To resolve these issues, we suggested a new protocol bridge system named TinyONet. In TinyONet, virtual sensors play roles as virtual counterparts of physical sensors and they dynamically group to make a functional entity, *Slice*. Instead of direct interaction with individual physical sensors, each sensor application uses its own WSN service provided by *Slice*s. If a new kind of service is required in TinyONet, the corresponding function can be dynamically added at runtime. Beside the data-centric communication, it also supports the node-centric communication and the synchronous access. In order to show the effectiveness of the system, we implemented TinyONet on an embedded Linux machine and evaluated it with several experimental scenarios.

## 1. Introduction

Wireless Sensor Networks (WSNs) have been considered as a key technology for a wide range of application domains that cannot be resolved with traditional wireless *ad hoc* networks [1, 2, 3]. WSNs are expected to be versatile in many situations [4], but no standards and design rules have been followed in designing sensor network architectures because every sensor application has its own unique purpose and requires various sensor hardware and network capability. For this reason, traditional WSN research projects tend to design their sensor applications depending on specific sensor hardware, network routing schemes, and operating systems. However, this monolithic and all-in-one approach forces a sensor application to be tightly coupled to a proprietary sensor network architecture and it results in several technical weaknesses; interoperability, extensibility, difficulty of development, and reusability of sensing data [5].

As an alternative method to resolve the issue, the protocol bridge research seamlessly integrating WSNs with the TCP/IP network has attracted researchers' attention [6, 7] including the IETF [8]. The main purpose of this research is to provide Internet users with a unified interface to transparently access sensor networks using the TCP/IP protocol. It is often argued whether the TCP/IP protocol stack and the node-centric communication are suitable for WSNs or not. However, if WSNs support the TCP/IP protocol, existing TCP/IP network applications can use heterogeneous WSNs as ordinary network participants and this seamless integration can be used on diverse applications such as a smart space or Next Generation Network (NGN).

In spite of promising advantages of the protocol bridge research, existing studies still have several issues that should be deeply discussed. Most of all, those studies give no consideration to the reusability of sensing data. Their main purpose is to ensure the end-to-end communication between sensors and external sensor applications. It means every session through a bridge is independent, regardless of the similarity of sensing data. Usually, in WSNs, a data reporting model can be categorized into four models: periodical, event-driven, query-driven, and hybrid [9]. If a single external sensor application uses a WSN, each model may show its own pros and cons. However, when multiple sensor applications share a WSN simultaneously, things begin to take on a new aspect. In this circumstance, a WSN using a periodical or an event-driven model has to repeatedly report the same data to multiple external applications. Even with a query-driven model, the same sensing data may be requested by multiple sensor applications and the data will be repeatedly transmitted as well. In WSNs, the transmission energy is much larger than the computational energy, so reducing the number of transmissions on WSNs is always an important design factor [3, 5], but the protocol bridge research has not seriously discussed the reusability of sensing data yet.

Another consideration is that the current studies do not provide the data-centric communication method. As indicated in [10], the data-centric method is very suitable for the characteristics of sensor networks. The data-centric method does not collect sensing data directly from each sensor's raw data but allocates abstract sensing tasks to the whole sensor network. This kind of communication method enables sensor applications to consider the entire sensor network as an abstract data infrastructure. Although the node-centric method is undoubtedly a basic method for WSNs, some sensor applications may need to access sensing data via the data-centric method.

To overcome the shortcomings of existing research, we proposed a new bridge system, TinyONet, in which virtual sensors cache sensing data and gather into functional entities that provide multiple sensor applications with both the data-centric and the node-centric communications. Most WSNs use a sink node as a portal to communicate with physical sensors on a sensing field. All data between sensors and external sensor applications must pass through the sink node. Moreover, the sink node is quite free for the limitation of computational and energy resources comparing to ordinary sensors. We paid attention to this feature and we anticipated that a proper caching scheme on the sink node could improve the reusability of sensing data and provide a sophisticated data access. For the node-centric communication, TinyONet prepares a bypass method with which sensor applications can detour TinyONet and directly communicate to physical sensors. We implemented TinyONet on an embedded Linux machine and made it cooperate with TinyOS-based sensors. If a new kind of data access service is needed, the service can be dynamically injected into a running sink node without disturbing other parts of the system.

The remainder of this paper is divided into five sections. Section 2 briefly analyzes the pros and cons of existing research and considers design principles. Section 3 describes a conceptual design, overall architecture, and basic ideas of TinyONet. Section 4 shows the implementation and operations of TinyONet. In Section 5, we evaluate TinyONet with the considered design principles and analyze the performance results. Finally, Section 6 concludes the paper.

## 2. Design Issues

### 2.1. Related Works

There has been a certain amount of research projects on a protocol bridge between sensor networks and the TCP/IP network. Lei *et al.* [6] classified these research projects into a Gateway-based approach and an Overlay-based approach. In the Gateway-based approach, there are application-level gateways [6, 11, 12] and a Delay Tolerant Network [13]. The application-level gateways have a role to translate protocols of both networks and to relay data packets. Since the protocol translation only occurs within the gateways, this approach can easily support heterogeneous sensor networks. However, the approach cannot offer a direct access to a specific sensor node. Unlike other gateway-based research, the Delay Tolerant Network proposed a Bundle Layer residing on both network sides and a gateway. This Bundle Layer hides the differences of protocols and it enables easy integration of heterogeneous sensor networks. However, the installation of the Bundle Layer can be a critical burden to resource-scarce sensors.

The overlay-based approach suggests overlaying protocol stacks on the sensor network [7, 8, 15] or the TCP/IP network [14]. 6LoWPAN [15] and u-IP [7] insist that additional protocol stacks on sensor nodes can resolve the integration problem and provide the end-to-end communication. However, this approach causes constant controversy because the installation of protocol stacks and the protocol translation overhead can go beyond typical sensor's capability. On the other hand, [14] proposed an installation of additional protocol stacks on the TCP/IP network. It has the same advantage like other overlay-based approaches, but it greatly hurts the consistency and the interoperability of the TCP/IP network.

In summary, existing research projects have mainly focused on providing the node-centric communication which enables the end-to-end communication between two heterogeneous networks. However, these all projects lack in efficient reuse of sensing data and a highly sophisticated data access based on the data-centric manner.

### 2.2. Design Principles

After investigating existing research, we could pick out several design principles to create a desirable protocol bridge as follows:

#### Reusability of Sensing Data

The reported sensing data from sensing fields should be reused when multiple external sensor applications use the same WSN. This data reuse can decrease the total number of transmission on WSNs and it will lead to the improvement of energy efficiency. It will also enable more rapid response to external sensor applications.

#### Providing Diverse Communication Methods

The designed system should provide both the data-centric and the node-centric communication methods. Usually, the data-centric method is well suited for the characteristics of WSNs [10], but the node-centric method is still required to integrate WSNs with the conventional TCP/IP network. This feature will permit an external sensor application to select its preferred communication method.

#### Dynamic Adoption of Various Sensing Data

Depending on the type of application domains, WSNs may maintain various kinds of sensing data such as sensor's physical location, velocity, or residual energy. However, in most cases, it is very difficult to extend the schema of sensing data after the system deployment. The proposed system should provide a method to easily adopt various sensing data without system redesign and modification.

#### Dynamic Service Extension at Runtime

A desirable system supporting the data-centric communication should provide an easy way to add new queries or functions dynamically when external sensor applications require new services. Undoubtedly, this service extension should be performed with on-the-fly manner not to disturb other running sensor applications and even not to cause system stop.

#### Providing Sophisticated Functional Components

The basic reuse of sensing data can improve the energy efficiency and the response time, but the cached sensing data could be utilized in various ways. In addition to simple functions such as returning the maximum value of the specific area's temperature, the target system should provide highly sophisticated functional components which can compose sensing data and deduce the meaningful information from the data.

## 3. System Design

### 3.1. Conceptual Design

**Figure 1.**
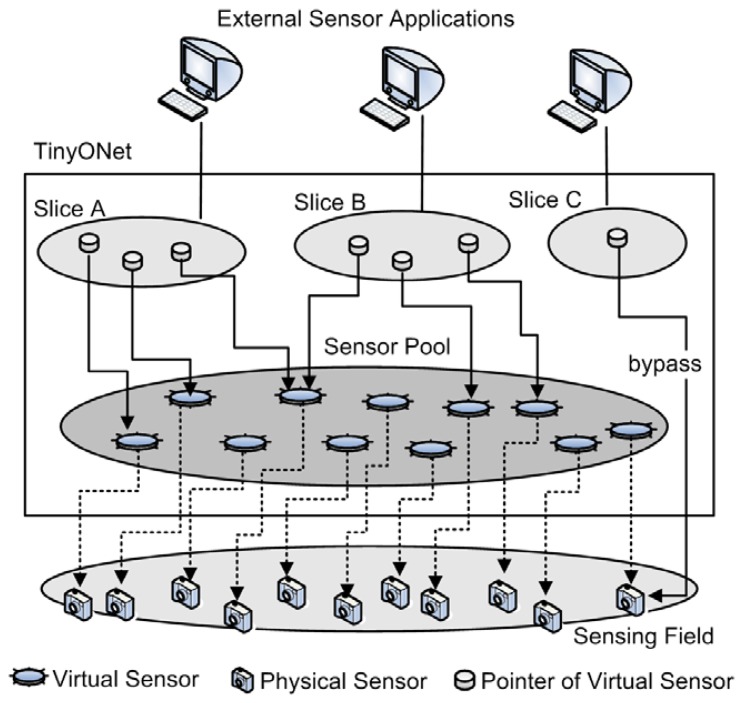
Conceptual design of TinyONet.

In general, a sink node occupies an important position in WSNs because all the data and commands have to pass through it. For this reason, the sink node is considered as an ideal place to translate protocols or perform some manipulations on sensing data. In our design, TinyONet is located on the sink node and maintains virtual sensors which perform as virtual counterparts [16] of physical sensors. A virtual sensor caches the latest sensing data from its corresponding physical sensor and behaves as a proxy of real sensor to external sensor applications. As shown in Figure 1, when an external sensor application issues a data-centric request, TinyONet dynamically organizes virtual sensors into a virtual functional entity named *Slice* which serves the request. The *Slice* has a socket connected to the requesting external sensor application and provides actual WSN services. By maintaining concurrent *Slice*s, TinyONet can provide different kinds of data access services to multiple sensor applications using the same cached data. For example, the virtual sensors' temperature data collected from a building can be repeatedly used to serve a large number of applications. The cached data can be simultaneously reused not only by many building residents' PDA for smart environment, but also by some building management applications such as wine reservation.

The proposed architecture is quite suitable for the periodic and the event-driven reporting models. In these models, the latest sensing data continuously flows into a virtual sensor when a new event occurs on a sensing field. It means that a cache miss will be resolved within a few seconds or a single period. However, the situation is somewhat different in the case of the query-driven model. In the query-driven model, when the query is delivered to a *Slice*, some virtual sensors' data can be out of date, comparing to physical sensors' current data. In order to resolve this issue, we designed the system to provide the synchronous access to physical sensors' current data when the query driven-model is needed. Besides, *Slice* should provide the node-centric communication method for sensor applications which need to directly communicate with physical sensors.

Although *Slice* has a role to interact with sensor applications, it is not desirable for *Slice* to have actual processing functions. If the processing code is written in a *Slice*, the *Slice* has to be replaced whenever a new service is needed. For this reason, we designed the system to offer a convenient method with which *Slice* can delegate its actual processing codes on-the-fly.

### 3.2. Architecture and Basic Ideas of TinyONet

**Figure 2.**
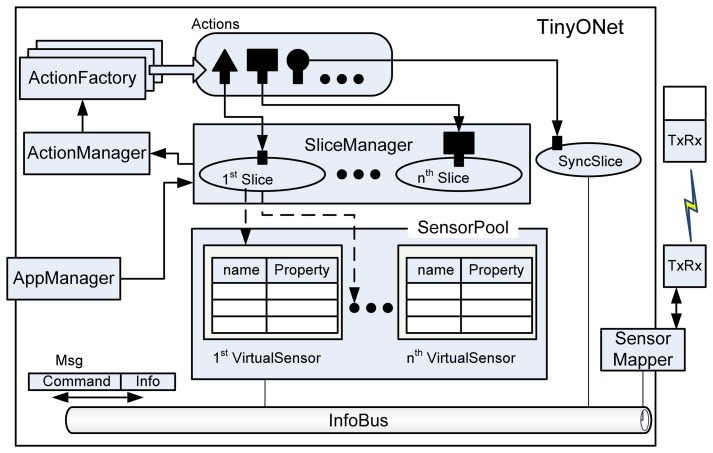
Components of TinyONet.

In order to realize the conceptual design and satisfy design principles, we designed several components with the Object-Oriented Design (OOD) methodology. As shown in Figure 2, TinyONet consists of many classes; *VirtualSensor*, *SensorPool*, *Slice*, *SyncSlice*, *SliceManager*, *AppManager*, subclasses of *Action*, subclasses of *ActionFactory*, *ActionManager*, *SensorMapper*, *InfoBus*, *Msg*, *Info*, subclasses of *Command*, *TxRx*, and *Property*.

#### Maintaining Sensing Data of Virtual Sensor

To provide the reusability of sensing data, *SensorPool* component spawns *VirtualSensor*s as the same number of physical sensors on a sensing field. In *SensorPool*, *VirtualSensor*s are running to cache the sensing data reported from their corresponding physical sensors. By using *TxRx* module, physical sensors communicate with TinyONet. Each *VirtualSensor* has a Map-based cache where the key is a property name and the value is the instance of *Property* class. According to its application domain, each individual sensor application may require a new kind of data such as sensor's position or residual energy. Since data fields are stored in the Map-based cache, *VirtualSensor* class can easily adopt new kinds of sensing data without ruining its internal structure.

#### Message Delivery between Components

Usually, it is not easy for external sensor applications to establish TCP/IP sessions with physical sensors because most WSNs use a non-IP protocol. Additionally, maintaining synchronous peer-to-peer sessions between virtual and physical sensors can cause an excessive communication and a computational overhead. For this reason, we designed the simple Message Oriented Middleware (MOM) [17], named *InfoBus*, using the Observer design pattern [18]. Several *SyncSlice*s, *SensorMapper*, and *SensorPool* exchange sensing data and commands through *InfoBus* using *Msg*, *Command* and *Info* classes. Unlike ordinary session-oriented communication, participants just throw packets into *InfoBus* instead of direct connection to other parties. Then, these packets are automatically and asynchronously delivered to the target party through *InfoBus*. This architecture simplifies the multiparty communication dramatically and it enables physical sensors and TinyONet components to easily exchange messages without considering the complex session handling.

#### Providing Customized WSN Services and Dynamic Service Extension

Since each *Slice* is designed as an independent thread, it can provide a customized WSN service to the requesting sensor application without disturbing other *Slice*s. In order to serve the customized WSN services, *SliceManager* creates a *Slice* which chooses various *Action*s to manipulate *VirtualSensor*s' data depending on the request. Instead of squeezing all functions into TinyONet, it delegates requested functions to external loadable modules, *Action* subclasses. With this mechanism, TinyONet serves external sensor applications that need various and unique WSN services for their own purposes. *AppManager* has a main role to communicate with external sensor applications and it delivers the requests from sensor applications to *SliceManager*. It also manipulates a dynamic function loading in cooperation with *ActionFactory* subclasses. By Using Strategy [19] and Abstract Factory [20] design patterns, *ActionFactory* can support dynamic addition of new functions which were not installed at deployment time.

#### Providing Synchronous Access and the Node-Centric Communication

Since *Slice* uses *VirtualSensor*s' cached data, it cannot use unreported data residing on physical sensors. In most situations, this may not be critical because a data reported from physical sensors takes only a few seconds and this update can be reported promptly if a proper *Action* is used. However, if an external sensor application needs to know the physical sensor's current data, TinyONet should provide synchronous access. Additionally, TinyONet should provide the node-centric communication for an application which needs to communicate directly to a physical sensor. For these diverse communication methods, *SyncSlice* is designed to provide both synchronous access and the node-centric communication. When these kinds of communication methods are requested, *SliceManager* spawns a *SyncSlice* to handle these requests. Unlike ordinary *Slice*, *SyncSlice* makes a session that promptly invokes a response from physical sensors.

## 4. Implementation and Operations

### 4.1. Prototype Implementation

The designed components can be implemented with contemporary Object-Oriented languages such as Java or C++. Although Java is safer and more convenient than C++ in most respects, C++ codes can be run on more platforms than Java because some platforms do not have their own Java Virtual Machine (JVM). Since we wanted for TinyONet to be ported on a wide range of platforms including resource-restricted embedded machine, C++ is selected as the implementation language. We also anticipated that C++ code can be easily converted into Java with a little effort.

**Figure 3.**
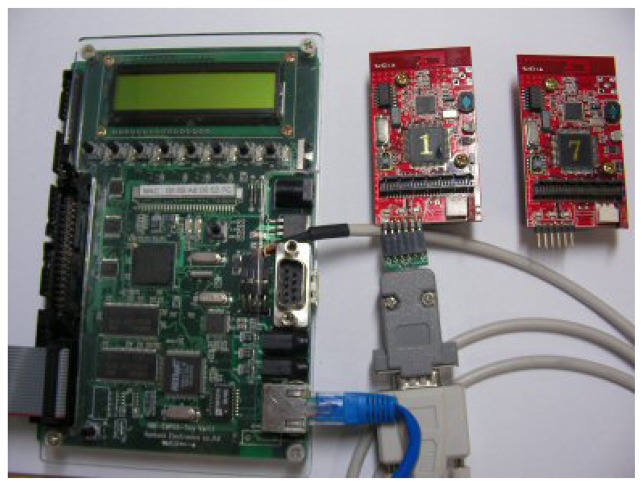
A Linux machine and sensors for the prototype implementation of TinyONet.

We implemented TinyONet on a commercial embedded Linux machine named EMPOS-Tiny [21], which consisted of Intel XScale PXA-255 CPU, 32 Mbyte Flash memory and 64 Mbyte RAM. A target mote is a commercial product, ZigbeX [22], based on ATMega-128 CPU that has 128 Kbyte code area, 4 Kbyte EEPROM and 2 Kbyte SDRAM. The mote runs TinyOS and uses ZigBee as RF communication. Each mote can sense temperature, humidity and intensity of illumination. All motes install the *TxRx* module for their communications. The hardware configuration is shown in Figure 3.

### 4.2. Maintaining Sensing Data of Virtual Sensor

**Figure 4.**
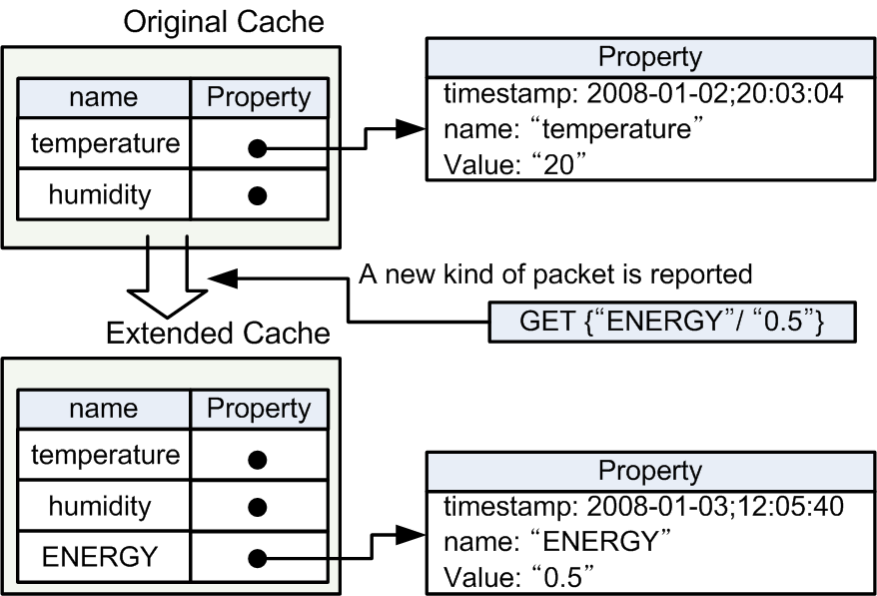
Cache structure of *VirtualSensor*.

Each *VirtualSensor* has a Map-based cache where the key is a property name and the value is the instance of a *Property* class. The *Property* class has a timestamp, a property name and value. If a physical sensor has a sensing ability of temperature and humidity, the corresponding virtual sensor will have two instances of the *Property* class as shown in the upper part of Figure 4.

As shown in the lower part of Figure 4, *VirtualSensor*'s cache can be dynamically extended when a reported data packet contains new kinds of properties even after deployment. For example, if a physical sensor which reports residual energy is newly spread on the existing sensing field, this sensor may report its residual energy periodically. After receiving the first report packet, the corresponding *VirtualSensor* will add a new property holding ‘residual energy’ which other existing *VirtualSensor*s' caches do not have. With this approach, *VirtualSensor* can dynamically adopt a new kind of property without the system modification or the system halt. This paper is limited in that the cache entry deletion was not implemented. We did not include cache entry deletion for two reasons. First, we anticipated that a physical sensor rarely removes the existing properties provided by its hardware after deployment. Second, we concluded that the aging of cache entries could be enough to complement the cache entry deletion. However, the function of the cache entry deletion may be needed for general purpose, therefore it would be added in the future study.

### 4.3. Message Delivery between Components

**Figure 5.**
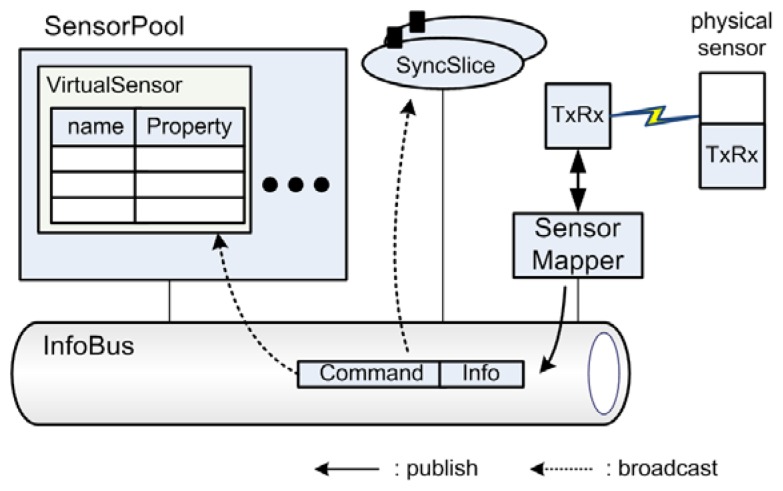
Message delivery through *InfoBus*.

As like other Observer design pattern [18] based MOMs, there are single Subject and multiple Observers in TinyONet. *InfoBus* is the subclass of the Subject class and it is responsible for broadcasting incoming messages to the registered Observers. *SensorPool*, *SensorMapper* and *SyncSlice*s are the subclasses of the Observer class and they listen to *InfoBus*. If a new data packet arrives at *SensorMapper* from a sensing field, the packet is packaged into a *Msg* and the *Msg* is published on *InfoBus*. Then, this *Msg* is delivered to *SensorPool* and the group of *SyncSlice*s. If *SensorPool* is the *Msg*'s target, it will parse the *Msg* and perform an ordered processing with *Command* and *Info* of the *Msg*. In the case of *SyncSlice*s, they promptly process the *Msg*s and perform the action on receiving *Msg*s. Conversely, if there are some data to be delivered to physical sensors, *SyncSlice*s or *SensorPool* will publish the data on *InfoBus*. Then, this data is handed over *SensorMapper* and then it is transmitted to physical sensors through *TxRx*. This message-oriented communication is depicted in Figure 5.

### 4.4. Packet Format on a Sensing Field

**Figure 6.**
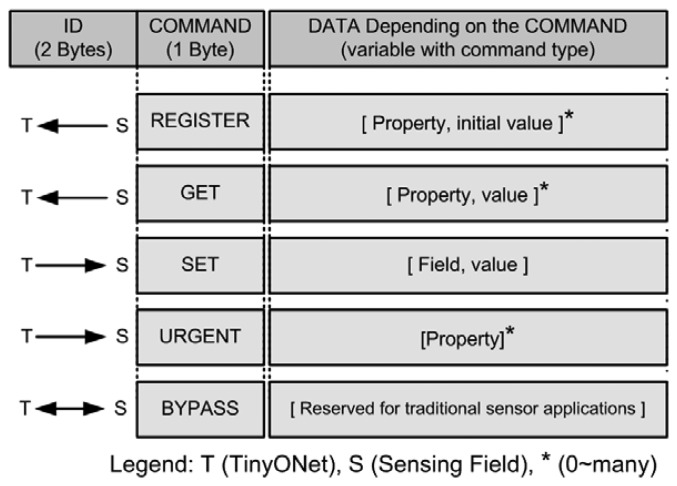
Structure of the data packet.

In TinyONet, several data packets are defined in order to communicate with physical sensors as shown in Figure 6. Each packet has ID, COMMAND and data fields. The ID field is two bytes-length field holding the unique identifier of an individual physical sensor. The COMMAND field has a single byte that represents packet types. There are five packet types; REGISTER, GET, SET, URGENT and BYPASS. The contents and length of a packet vary with the type of the COMMAND.

The REGISTER and GET packets are transmitted from a sensing field. When a sensor is newly launched onto a sensing field, it will send a REGISTER packet to *SensorMapper*. When this packet is delivered to *SensorPool* from *SensorMapper*, it will create an instance of *VirtualSensor*. A GET packet is sent when a sensor catches a new sensing data to report. This reported data in the GET packet will be stored into the corresponding *VirtualSensor*'s cache with a timestamp. The SET and URGENT packets are used to send commands from TinyONet to the sensing field. The SET data packet usually contains some commands for physical sensors. The basic communication model of TinyONet is based on asynchronous reporting, but the synchronous access is also required to support the query-driven reporting model. For this purpose, the URGENT packet is used and this is presented in section 4.8. The BYPASS packet is a special bidirectional packet that is not processed within TinyONet. When some sensor applications need to use the node-centric communication, they can use the BYPASS packet to deliver their proprietary data to the sensors and vice versa. A detailed procedure for BYPASS and URGENT packets is described in section 4.8.

### 4.5. Communication between External Sensor Applications and Physical Sensors

Up to now, most WSNs adopted their own proprietary network routing protocols instead of using IP and TCP/IP protocol stack because an authorized standard for WSN address scheme has not been established yet. In TinyONet, each sensor node is allocated a 16 bit address spanning from 0 ∼ FFFE and FFFF is reserved for the broadcast address. A TinyOS-based mote of our research, ZigbeX [22], uses TOS_Msg format [23] over ZigBee radio channel for its data communication. In order to transmit a packet, each mote composes its data packet with its ID, a command, and command dependent data as shown in Figure 6, and then it puts the data packet in the payload of TOS_Msg. Since ZigBee uses a wireless channel, a transmitted TOS_Msg packet will be arrived at every mote. However, only a mote having the same ID of the packet will accept the packet. Each mote examines the ID of the transmitted packet and decides whether to drop or accept. If the ID of the packet is matched to its own ID, the mote will accept it. Exceptionally, a mote operating as a base station listens to every packet and delivers the arrived packet to its connected *SensorMapper*, which decides whether to drop or accept. Although this proposed WSN protocol provides simple functions, this protocol can be easily extended or replaced without affecting TinyONet itself.

**Figure 7.**
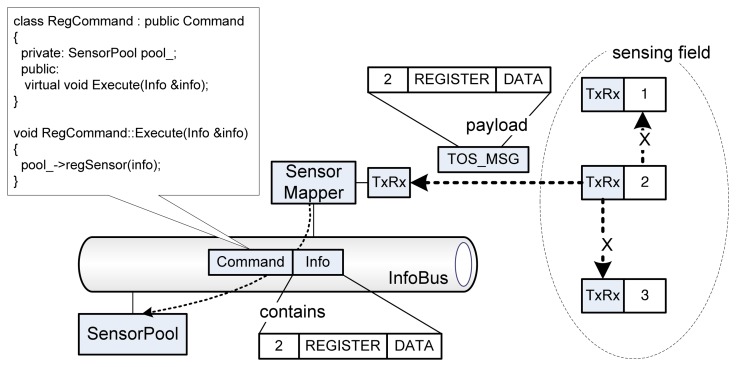
Processing of a REGISTER packet transmitted from a sensing field.

A packet transmission can be started from the sensing field or external applications. From the sensing field, one of the REGISTER, GET, or BYPASS packet is reported and the packet is served by corresponding *Command* subclasses; *RegCommand*, *GetCommand*, and *ByRcvCommand.* As shown in Figure 7, if a sensor transmits a REGISTER packet containing its ID, the arrived packet on *SensorMapper* will trigger to make a *RegCommand* and an *Info* class instances. Since the *RegCommand* class inherits the *Command* class, the delivered *RegCommand*'s command for creating new sensor (i.e. regSensor() function) will be executed from the target class, *SensorPool*. The payload of TOS_Msg (i.e. the sensor's original packet) is stored into an *Info* class instance and the instance is used by the concrete *Command* class instance, *RegCommand*. This packet processing based on Command design pattern [24] can provide an advantage of extensibility. If a new kind of packet is required, TinyONet just adds the corresponding inherited *Command* class which can handle the packet. Undoubtedly, this pattern enables smooth extensibility without ruining other parts of the system.

**Figure 8.**
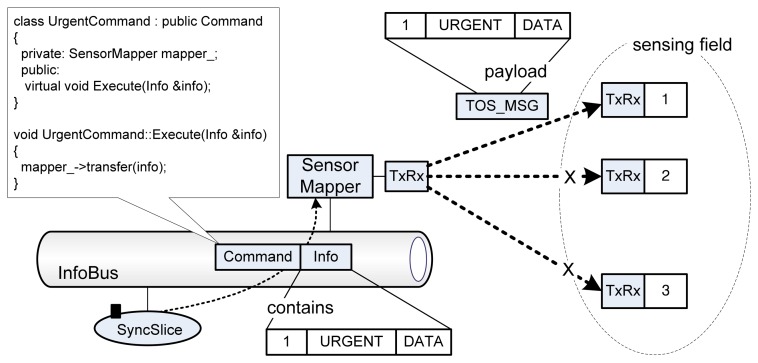
Processing of a URGENT request which accesses to a specific sensor node.

When an external sensor application needs to access specific sensor nodes, it can issue a request that invokes a BYPASS or an URGENT packet. The request is served a *SyncSlice* and then the *SyncSlice* creates an instance of *Command* subclasses; *BySendCommand* for the BYPASS and *UrgentCommand* for the URGENT respectively. As shown in Figure 8, if an URGENT packet is needed, a *SyncSlice* prepares an *UrgentCommand* and an *Info* class instances. The *UrgentCommand* contains a command that will be executed at *SensorMapper* and the *Info* instance contains the target sensor ID, URGENT command, and the related data. When the *UrgentCommand* is delivered to *SensorMapper*, the *UrgentCommands* command for *SensorMapper's* transferring code is executed. The code makes the URGENT packet with the contents of the *Info* instance. Then, the URGENT packet is delivered to the target sensors on the sensing field. As described above, the only target sensor having the same sensor ID will accept the URGENT packet while other sensor nodes will drop the packet.

### 4.6. Customized WSN Services

When an external sensor application issues a request, it will send a request message to *AppManager* as follows:

[OP][OP_ARGUMENT]*(*means0∼many)

**Figure 9.**
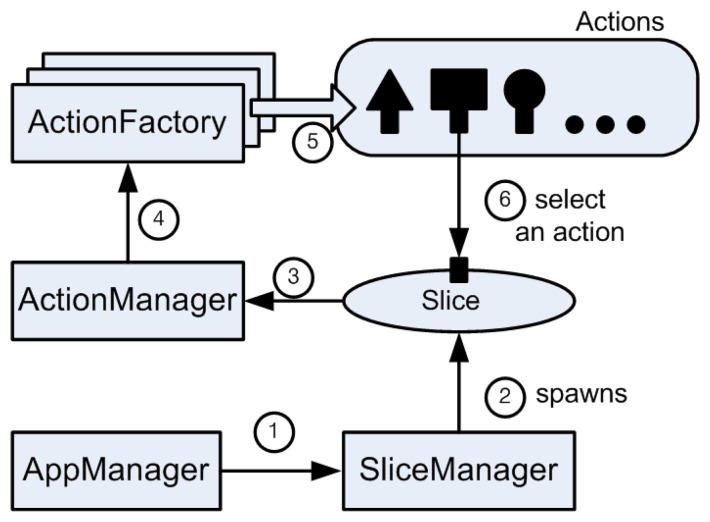
Process of *Slice* creation.

The [OP] field indicates a requested operation which *Slice* will serve, such as calculating the maximum value of sensing data. The [OP_ARGUMENT] fields are variable depending on the [OP] field. When the request is arrived at the server socket of *AppManager*, it delivers the request to *SliceManager. SliceManager* spawns a new *Slice* and it hands over the connected socket and the request message to the *Slice*. Then, the *Slice* chooses a proper *Action* to serve the requested [OP] in cooperation with *ActionManager*. After selecting an *Action*, the *Slice* orders the selected *Action* to process [OP_ARGUMENT] fields. This procedure is depicted in Figure 9.

*Slice* can freely choose different actions depending on the type of request. The mechanism is based on the Strategy design pattern [19]. In this pattern, a main class can replace a strategy class according to situations. The adoption of the Strategy pattern provides two considerable advantages. First, *Slice* class can provide different actions without its modification. It just chooses a proper *Action* and delegates the *Action* to serve an actual process. Second, there is no restriction on the argument format because the corresponding *Action* is only responsible for understanding its argument. For example, the following MAX and REPORT requests may require different arguments, but *Slice* is not necessary to understand the detail format. *Slice* can handle all the requests in the same way because each corresponding *Action* takes whole charge of handling its argument. These advantages enable third-party users to make and add customized services without affecting other parts of the system.

$$\begin{array}{c} \left[\text{MAX}][\text{left}=\text{5},\text{top}=\text{10},\text{right}=50,\text{bottom}=50][\text{temperature}\right]\\ \left[\text{REPORT}][\text{humidity}\right] \end{array}$$

### 4.7. Dynamic Service Extension at Runtime

Undoubtedly, the Strategy design pattern enables TinyONet to extend its services without disturbing other parts of the system, but the pattern is effective only when the system is recompiled at the source code level. That is, the adoption of the Strategy pattern is not sufficient to guarantee the dynamic addition of new services at runtime, therefore other technical methods should be considered to support the dynamic service extension.

Usually, it is difficult to dynamically extend the compiled C++ code, because a developer cannot know the name of class to be made after the development phase. For example, if an ‘average’ action is needed, a developer may want to write codes as like this:

Action*pAction=new Average Action();

However, this coding is nearly impossible because the developer cannot know the name of the target class, ‘AverageAction’, in advance. Needless to say, the AverageAction class will be developed after the code is released.

**Figure 10.**
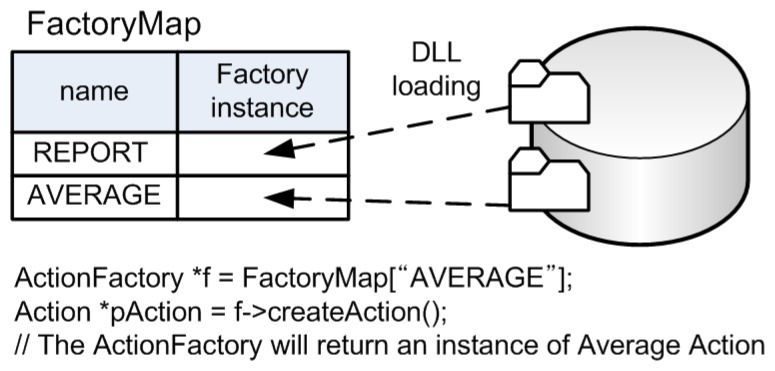
Dynamic action loading with the Abstract Factory pattern and the C++ dynamic loading.

We overcame the issue by combining the Abstract Factory pattern [20] with the C++ dynamic loading [25]. The C++ dynamic loading enables the compiled code to use the classes in Dynamic Linked Library (DLL). However, this binary level extension also has the same weakness as described above. In the case of using the C++ dynamic loading, a developer still has to know the target class name in advance. The Abstract Factory pattern can be a missing part of the solution to the problem. As shown in Figure 10, each Factory class is dynamically loaded from DLLs and it is stored into the Map data structure with its action's name as the key. When an action is requested, the corresponding Factory class is retrieved from the Map and then the Factory class creates the instance of requested action. Using this combining method, the dynamic service extension at runtime is possible without indicating class names in the source code.

### 4.8. Providing Synchronous Access and the Node-Centric Communication

If a physical sensor receives an URGENT packet, it promptly reports the requested sensing data using a GET packet. When an URGENT packet is used, *SyncSlice* is allocated instead of the ordinary *Slice*. Since the *SyncSlice* class is inherited from both Observer and *Slice* classes, it can act as not only *Slice*, but also the Observer. It means that the GET packet fired by the URGENT packet is directly delivered to the corresponding *SyncSlice* and then the *SyncSlice* promptly reacts to process the packet. With this synchronous access mechanism, external sensor applications can get the latest data in the case of the query-driven reporting model. Additionally, the GET packet is also delivered to the *SensorPool* and it is used to update the corresponding *VirtualSensor*'s data for efficiency.

When an external sensor application needs to bypass TinyONet and directly communicate with physical sensors, it can use the BYPASS request as follows:

[BYPASS][BYPASS_custom data]

When TinyONet receives this request, it allocates a *SyncSlice* to handle the BYPASS request and then the *SyncSlice* sends the [BYPASS_custom data] to the target physical sensor using a BYPASS packet. Reversely, if a physical sensor needs to bypass TinyONet, the sensor can also use a BYPASS packet. If a BYPASS packet is transmitted from physical sensors, it is delivered to the listening *SyncSlice* that is connected to the requesting external sensor application. Then, the *SyncSlice* just relays the packet's data to the application. During the process, the *SyncSlice* does not touch but just relay the data between two networks. Comparing to the synchronous access using the URGENT/GET packets, a BYPASS packet does not update *VirtualSensor*'s data because the BYPASS packet is designed to provide a direct passage that detours around TinyONet.

## 5. Experiments and Evaluation

In order to make an experimental sensing field, we deployed ten sensor motes. One mote is connected to TinyONet as the base station of the sensing field and the remained nine motes are deployed in three rooms. We have located three motes in each room's corner in order to evaluate the effectiveness of TinyONet. We conducted two experiments to examine the response time and the accumulated number of generated packets on WSN.

### 5.1. Response Time

In the first experiment, external sensor applications concurrently gathered the temperature data of three rooms with two cases; a conventional WSN and TinyONet. To examine the response time of concurrent applications, we performed the test ten times by increasing the number of sensor applications one by one. To emulate conventional WSN, the URGENT and GET packets were used to provide the synchronous communication. For the case of TinyONet, the REGISTER and GET packets were used as usual.

Response time, *T_response_*___*_time_*, is defined as the amount of time that the last sensor application takes to get the response when several external sensor applications issue a request simultaneously. As shown in Equation (1), *T_response_*___*_time_* is composed of three factors; *T_transmission_*, *T_process_*, and *T_wsn_. T_transmission_* is the transmission time between a sensor application and TinyONet. *T_process_* is the processing time within TinyONet. *T_wsn_* is the elapsed time to complete gathering sensing data on WSN.

(1) $$T_{\text{response}\_\text{time}}=T_{\text{transmission}}+T_{\text{process}}+T_{\text{wsn}}$$

**Figure 11.**
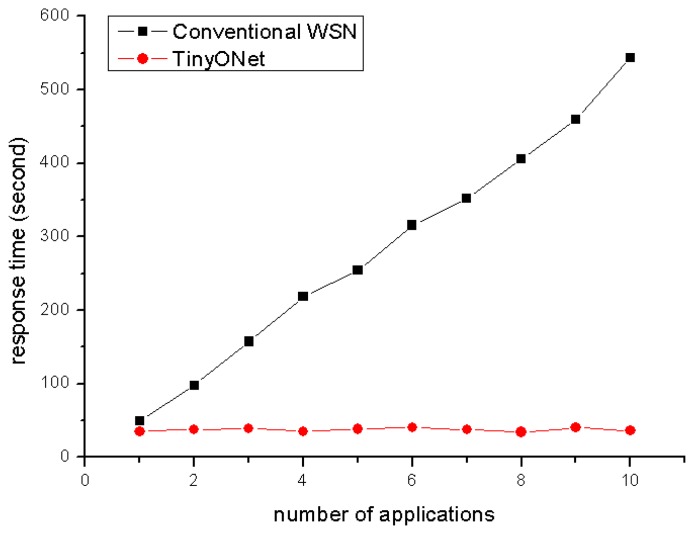
Response time as increasing the number of applications.

The graph in Figure 11 shows the response time of two cases. The graph indicates that the response time of the conventional WSN is linearly increased according to the number of sensing applications. Comparing to this, the response time of TinyONet is nearly constant value regardless of the number of sensor applications. We identified that the difference is caused from *T_wsn_* . Both cases have similar *T_transmission_* and *T_process_*, but they have totally different *T_wsn_* . In ordinary WSN, only a single packet can be transmitted at a time over radio due to the lack of the channel multiplexing. That is, multiple requests from concurrent sensor applications have to be queued and processed one by one on WSNs. For this reason, in the conventional WSN, the response time of the n-th sensor application, *T_response_*___*_time_* (*n*), can be modeled as Equation (2).

(2) $$T_{\text{response}\_\text{time}}\;(n)=T_{\text{transmission}}\;(n)+T_{\text{process}}\;(n)+\sum_{k=1}^{n}T_{\text{wsn}}\;(k)$$

While the *T_wsn_* of the conventional WSN is affected by the number of sensor applications, the *T_wsn_* of TinyONet is nearly regular because the elapsed time for completing the report session is independent of the number of external sensor applications. Since the data packets on WSN are asynchronously transmitted independent of the external sensor applications' request, *T_wsn_* is considered as a constant value. Therefore, the response time of the n-th sensor application in TinyONet can be modeled as Equation (3). As shown in the equation, all the applications share the same elapsed time, *T_wsn_*___*_common_* for communicating with WSN.

(3) $$T_{\text{response}\_\text{time}}\;(n)=T_{\text{transmission}}\;(n)+T_{\text{process}}\;(n)+T_{\text{wsn}\_\text{common}}$$

Generally, *T_transmission_* and *T_process_* are much smaller than *T_wsn_*___*_common_*, therefore the response time of TinyONet seems a constant value regardless of the number of sensor applications.

### 5.2. Accumulated Number of Generated Packets

The purpose of the second experiment was to examine the number of generated packets on WSN when different data update cycles are given. Since the data update cycle means periodic time for fetching latest data from WSNs, a request interval of conventional WSN and a reporting cycle of TinyONet can be considered as the data update cycle. In the case of the conventional WSN, a single sensor application is used to issue a request to each sensor by changing the request interval of 60, 120, or 180 seconds respectively. If the sensor application selects the request interval of 120 seconds, each sensor will receive a request at every 120 seconds. For TinyONet, a single sensor application was connected to TinyONet, which changed the sensor's reporting cycle of 60 or 120 seconds. We also added a random reporting cycle to the case of TinyONet. The random reporting cycle emulates a realistic sensing environment where sensor nodes report only if they have the sensing data to report. The range of random reporting cycle spanned from 30 seconds to 600 seconds.

We made the *TxRx* module of TinyONet record all incoming and outgoing packets regardless of its transmission success. The packet on the *TxRx* module is stored in the TOS_Msg format [23]. The message format consists of seven bytes header, payload and two bytes CRC (Cyclic Redundancy Check). The TinyONet packet is stored in the payload of the TOS_Msg. In the case of the conventional WSN, a BYPASS packet (17 bytes) was used to broadcast data gathering message and to transmit a request packet to a specific sensor node. Each sensor node also used the same BYPASS packet to report its sensing data. In the case of TinyONet, each sensor node used a REGISTER packet (23 bytes) to register itself and a GET packet (17 bytes) to report its sensing data.

**Figure 12.**
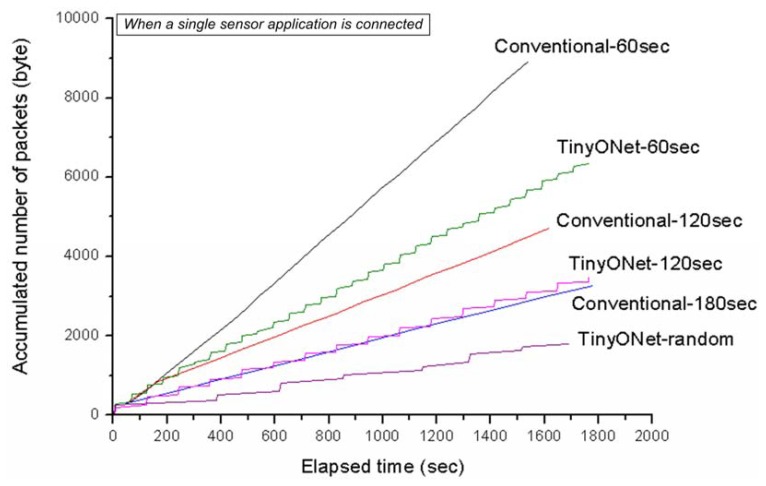
Accumulated number of packets on the *TxRx*.

Undoubtedly, the shorter data update cycle will generate more data packets on WSN. In Figure 12, we can see the fact that the packet number of ‘Conventional-60 sec’ exceeds that of ‘Conventional-120 sec’ and ‘Conventional-180 sec’. Although the shorter data update cycle generates more packets, it lets external sensor applications and virtual sensors have more recent data. For the same data update cycle, the generated packets of TinyONet are much less than those of conventional WSN. While a single session of conventional WSN is composed of a request and a response phases, a reporting phase solely consists of a single session in TinyONet. In view of the data update, fewer packets of TinyONet can make the same effect of data update in the conventional WSN. In addition, Figure 12 shows that TinyONet having the random reporting cycle generates much fewer packets on WSN. It means TinyONet's performance can be much better under the realistic sensing environment.

### 5.3. Evaluation

We evaluated the TinyONet in two aspects; the performance results and the design principles. Additionally, we will discuss the future study of TinyONet.

#### Performance Evaluation

In the first experiment, the response time of conventional WSN linearly increased in proportion to the number of applications, but TinyONet maintained the regular response time. It showed the reuse of sensing data with the cache structure can greatly improve the response time especially when a large number of applications share the same sensor network. The second experiment indicated that conventional WSN generated much more packets than TinyONet when the same data update cycle was given. The fewer packet generation in a sensing field will result in the energy saving of each sensor node and the improvement of radio channel availability.

#### Reusability of Sensing Data

TinyONet is designed to support the reusability of sensing data. For example, if a heater raises the temperature of a room, the temperature data is cached on its corresponding virtual sensor. Then, the cached sensing data can be repeatedly used to serve a lot of sensor applications without performance degradation. The experiments in Sections 5.1 and 5.2 proved that the reusability of sensing data is greatly useful for the improvement of the response time and reducing the packet transmission.

#### Providing Diverse Communication Methods

In the experiment in Section 5.2, TinyONet showed that it can support both the node-centric and the data-centric communication methods. Mainly, TinyONet is designed to support the data-centric communication, but it also enable conventional WSN applications to use the node-centric communication with a BYPASS request. The experiment of Section 5.1 showed that TinyONet can also support the synchronous access.

#### Dynamic Adoption of Various Sensing Data

To satisfy diverse sensor applications' requirements, TinyONet is designed to dynamically adopt various kinds of sensing data. To verify this feature, we located a new kind of physical sensor and ordered it to report its location and energy. Then, we checked whether the corresponding *VirtualSensor* to handle new properties well and to report the properties to external sensor applications as usual. The result was successful.

#### Dynamic Service Extension at Runtime

To show the feasibility of the dynamic service extension, we made a new *Action* class calculating an average value and we uploaded it to the running TinyONet. After uploading, we checked that an external sensor application could request the uploaded ‘average’ action for the average value of the first room's temperature. The result was successful. Since the ‘average’ action is not bundled at deployment time, we can verify that the dynamic service extension at runtime is successfully performed.

#### Providing Sophisticated Functional Components

To verify that TinyONet can provide highly sophisticated functional components, we made a ‘FireAlaram’ *Action* class. The *Action* checked the temperature of the same room and notified an alarm when all three motes simultaneously reported an abnormal high temperature. After uploading this *Action*, we let physical sensors in the same room report very high temperature. The *Action* handled this reporting as we expected. It shows TinyONet can load various services not only for simple reporting functions, but also for complex and sophisticated functions. Theoretically, third-party users can make any kind of *Action* class if a new *Action* class inherits the original *Action* class.

#### Future Study

In spite of the remarkable results of this research, several design issues are still remained to be deeply considered. The first issue is a security risk related to the dynamic service extension. Although the dynamic service extension is very useful, it makes system be vulnerable as [26] indicated. To resolve the issue, the code signing or secure uploading channel has to be implemented. Although this feature is not considered yet in the current implementation, we have a plan to add the feature to the next version of the system. Another issue is the cooperation of multiple TinyONets. If several virtual sensors on different TinyONets can cooperate and fuse their sensing data, external sensor applications can treat virtual sensors as the normal computing resources, just as the current GRID technology does. We think that this issue is noteworthy for studying intensively.

## 6. Conclusions

Recently, in the WSN research field, protocol bridge projects have been rapidly gaining researchers' attention. The previous research has focused on providing external sensor applications with the transparent end-to-end communication in the node-centric manner. This protocol bridge has been expected to trigger a variety of application domains, but existing studies have several issues to be seriously discussed, especially the reusability of sensing data and the support of the data-centric communication.

To resolve these issues, we suggested a new protocol bridge system called TinyONet which maintains virtual sensors as a virtual counterpart of physical sensors. In TinyONet, virtual sensors cache the reported sensing data from their corresponding physical sensors and dynamically group themselves into a functional entity named *Slice* which provides various WSN services. Using this mechanism, TinyONet can serve various and heterogeneous requests from multiple sensor applications with the cached sensing data. As a result, the response time is improved and the number of communication packets on WSN is reduced. In TinyONet, if a new service is required, it can be dynamically added without stopping the system. It can also support the node-centric communication for traditional sensor applications and the synchronous access for the query-driven reporting model. This research shows that the proper caching architecture in the protocol bridge can improve the communication efficiency of WSNs and can provide versatile data access methods as well.
